# Correction: Shape-controlled synthesis of micro-/nanosized Cu particles with spherical and polyhedral shapes using the polyol process

**DOI:** 10.1039/d4ra90091j

**Published:** 2024-09-10

**Authors:** Nguyen Thi Nhat Hang, Yong Yang, Le Hong Phuc, Nguyen Huu Tri, Ho Van Cuu, Nguyen Viet Long

**Affiliations:** a Institute of Applied Technology, Thu Dau Mot University 6 Tran Van On, Phu Hoa Ward Thu Dau Mot City 820000 Vietnam; b State Key Laboratory of High-Performance Ceramics and Superfine Microstructures, Shanghai Institute of Ceramics, Chinese Academy of Sciences 1295 Dingxi Road Shanghai 200050 China; c National Institute of Applied Mechanics and Informatics, Vietnam Academy of Science and Technology 291 Dien Bien Phu Ho Chi Minh City 700000 Vietnam; d Faculty of Natural Sciences Pedagogy, Saigon University 273 An Duong Vuong, Ward 3, District 5 Ho Chi Minh City 70000 Vietnam; e Faculty of Electronics and Telecommunications, Saigon University 273 An Duong Vuong, Ward 3, District 5 Ho Chi Minh City 700000 Vietnam nguyenvietlong@sgu.edu.vn

## Abstract

Correction for ‘Shape-controlled synthesis of micro-/nanosized Cu particles with spherical and polyhedral shapes using the polyol process’ by Nguyen Thi Nhat Hang *et al.*, *RSC Adv.*, 2024, **14**, 22403–22407, DOI: https://doi.org/10.1039/D4RA03643C.

The authors regret that the affiliation for Nguyen Huu Tri was incorrectly shown in the original manuscript. The corrected list of affiliations is as shown here.

In addition, the author contributions were incorrectly given. The corrected author contributions are given below.

## Author contributions

N. V. L. conducted the experiments, collected the data and results, wrote the entire original manuscript, and discussed the results and insights into the polyol processes for the synthesis of Cu nanoparticles (Author contribution: 90%). Y. Y. (Author contribution: 2%), L. H. P. (Author contribution: 2%), N. H. T. (Author contribution: 2%), N. T. N. H. (Author contribution: 2%), and H. V. C. (Author contribution: 2%) discussed experimental data and research results.

The Royal Society of Chemistry apologises for these errors and any consequent inconvenience to authors and readers.

